# Supplementary material for: Clinical Factors Associated with Reinfection versus Relapse in Infective Endocarditis: Prospective Cohort Study
Source: J Clin Med. 2021 Feb 13;10(4):748. doi: 10.3390/jcm10040748 (PMC7918007; doi:10.3390/jcm10040748)
Supplement: Supplementary file 1 [file jcm-10-00748-s001.pdf]

Table 1S. Characteristics of the index episode of infectious endocarditis depending on whether the species causing the recurrence was the same or different from the initial episode.

	Same species (N=60)	Different species (N=70)	P
Age (years)	67 (52 - 78)	64 (45 - 73)	0.099
Male gender	41 (68.3)	47 (67.1)	0.885
Hospital-acquired	30 (50.0)	45 (64.2)	0.142
Non-nosocomial healthcare related	23 (38.3)	14 (20.0)	0.019
Community-acquired	5 (8.3)	9 (12.8)	0.585
Diabetes mellitus	18 (30.0)	14 (20.0)	0.187
Coronary disease	17 (28.3)	20 (28.6)	0.555
Peripheral arterial disease	4 (6.7)	5 (7.1)	0.915
Cerebrovascular disease	14 (23.3)	8 (11.4)	0.071
Previous renal failure	12 (20.0)	11 (15.7)	0.523
~~Chronic liver disease~~Liver cirrhosis	8 (13.6)	13 (18.6)	0.442
Injected drug user	4 (6.7)	4 (5.7)	0.552
Neoplasia	7 (11.7)	15 (21.4)	0.129
Age-adjusted Charlson Comorbidity Index (points)	5 (2 - 7)	4 (1 - 5)	0.109
Site of infection			
Native valve	30 (50.0)	37 (52.9)	0.745
Prosthetic valve	20 (33.3)	27 (38.6)	0.535
Cardiac device	10 (16.6)	6 (8.6)	0.257
Involved valve			
Mitral	30 (50.0)	39 (55.7)	0.515
Aortic	21 (35.0)	31 (44.3)	0.281
Tricuspid	4 (6.7)	2 (2.9)	0.414
Pulmonary	2 (3.3)	1 (1.4)	0.595
Microbiology			
Gram-positive bacteria			
Coagulase-negative staphylococci	8 (13.3)	14 (20.0)	0.312
S. aureus	16 (26.7)	6 (8.6)	0.006
Enterococcus spp	17 (28.3)	8 (11.4)	0.015
Streptococcus spp	11 (18.3)	19 (27.1)	0.235
Gram-negative bacilli	4 (6.7)	5 (7.1)	0.957
Anaerobic bacteria	0	4 (5.7)	0.124
Candida	0	1 (1.4)	0.538
Polymicrobial	0	1 (1.4)	0.538
Other microorganisms	5 (8.3)	13 (18.6)	0.127
Negative cultures	2 (3.3)	9 (12.9)	0.063
Septic shock	2 (3.3)	3 (4.3)	0.778
Persistent bacteremia	9 (15.0)	6 (8.6)	0.265
CNS vascular events	7 (11.7)	12 (17.1)	0.378
Embolism	17 (28.3)	11 (15.7)	0.081
Heart failure	14 (23.3)	29 (41.4)	0.025
New or worsening renal insufficiency	20 (33.3)	19 (27.1)	0.443
Echocardiographic findings			
Vegetation	37 (61.7)	49 (70.0)	0.317
Perivalvular abscess	5 (8.3)	12 (17.4)	0.194

Valve perforation or rupture	6 (10.0)	8 (11.6)	0.772
Pseudoaneurysm	1 (1.7)	3 (4.3)	0.442
Intracardiac fistula	0	3 (4.3)	0.171
Surgical indication	25 (41.7)	50 (71.4)	0.002
Surgery performed	14 (23.3)	42 (60.0)	<0.01
Surgery indicated but not performed	11 (18.3)	8 (11.4)	0.268
Duration of antibiotic treatment	38 (26 - 53)	42 (28 - 53)	0.727
Time up to second episode	101 (60 - 349)	261 (102 - 781)	0.001

CNS: central nervous system. Quantitative variables are reported with median and interquartile range.

Table 2S. Microbiology of the first (y-axis) and second episode (x-axis) of IE in cases of reinfection by a different microorganism.

Microorganisms 1st episode	Microooganisms 2nd episode						
	Streptococcus spp	*Enterococcus* spp	*Staphylococcus* spp	Other bacteria	Yeast	Unknown[1]	Total
Streptococcus spp	5	7	2		1	4	19
Enterococcus spp	1	2	5				8
Staphylococcus spp	7	3	1	1	2	6	20
Other bacteria	2	5	3	3			13
Yeast			1				1
Unknown [1]	1	2	5		1		9
Total	16	19	17	4	4	10	70

In some cases, the bacteria causing the infection was a different species belonging to the same genus. [1]
In some cases, only the microorganism causing one of the episodes of EI was known

Appendix

Members of GAMES: Hospital Costa del Sol, (Marbella): Fernando Fernández Sánchez, Mariam Noureddine, Gabriel Rosas, Javier de la Torre Lima; **Hospital Universitario de Cruces**, (Bilbao): Roberto Blanco, María Victoria Boado, Marta Campaña Lázaro, Alejandro Crespo, Josune Goikoetxea, José Ramón Iruretagoyena, Josu Irurzun Zuazabal, Leire López-Soria, Miguel Montejo, Javier Nieto, David Rodrigo, Regino Rodríguez, Yolanda Vitoria, Roberto Voces; **Hospital Universitario Virgen de la Victoria**, (Málaga): Mª Victoria García López, Radka Ivanova Georgieva, Guillermo Ojeda, Isabel Rodríguez Bailón, Josefa Ruiz Morales; **Hospital Universitario Donostia-Policlínica Gipuzkoa**, (San Sebastián): Ana María Cuende, Tomás Echeverría, Ana Fuerte, Eduardo Gaminde, Miguel Ángel Goenaga, Pedro Idígoras, José Antonio Iribarren, Alberto Izaguirre Yarza, Xabier Kortajarena Urkola, Carlos Reviejo; **Hospital General Universitario de Alicante**, (Alicante): Rafael Carrasco, Vicente Climent, Patricio Llamas, Esperanza Merino, Joaquín Plazas, Sergio Reus; **Complejo Hospitalario Universitario A Coruña**, (A Coruña): Nemesio Álvarez, José María Bravo-Ferrer, Laura Castelo, José Cuenca, Pedro Llinares, Enrique Miguez Rey, María Rodríguez Mayo, Efrén Sánchez, Dolores Sousa Regueiro; **Complejo Hospitalario Universitario de Huelva**, (Huelva): Francisco Javier Martínez; **Hospital Universitario de Canarias**, (Canarias): Mª del Mar Alonso, Beatriz Castro, Teresa Delgado Melian, Javier Fernández Sarabia, Dácil García Rosado, Julia González González, Juan Lacalzada, Lissete Lorenzo de la Peña, Alina Pérez Ramírez, Pablo Prada Arrondo, Fermín Rodríguez Moreno; **Hospital Regional Universitario de Málaga**, (Málaga): Antonio Plata Ciezar, José Mª Reguera Iglesias; **Hospital Universitario Central Asturias**, (Oviedo): Víctor Asensi Álvarez, Carlos Costas, Jesús de la Hera, Jonnathan Fernández Suárez, Lisardo Iglesias Fraile, Víctor León Arguero, José López Menéndez, Pilar Mencia Bajo, Carlos Morales, Alfonso Moreno Torrico, Carmen Palomo, Begoña Paya Martínez, Ángeles Rodríguez Esteban, Raquel Rodríguez García, Mauricio Telenti Asensio; **Hospital Clínic-IDIBAPS, Universidad de Barcelona**, (Barcelona): Manuel Almela, Juan Ambrosioni, Manuel Azqueta, Mercè Brunet, Marta Bodro, Ramón Cartañá, Carlos Falces, Guillermina Fita, David Fuster, Cristina García de la Mària, Delia García-Pares, Marta Hernández-Meneses, Jaume Llopis Pérez, Francesc Marco, José M. Miró, Asunción Moreno, David Nicolás, Salvador Ninot, Eduardo Quintana, Carlos Paré, Daniel Pereda, Juan M. Pericás, José L. Pomar, José Ramírez, Irene Rovira, Elena Sandoval, Marta Sitges, Dolors Soy, Adrián Téllez, José M. Tolosana, Bárbara Vidal, Jordi Vila; **Hospital General Universitario Gregorio Marañón**, (Madrid): Iván Adán, Javier Bermejo, Emilio Bouza, Daniel Celemín, Gregorio Cuerpo Caballero, Antonia Delgado Montero, Ana García Mansilla, Mª Eugenia García Leoni, Víctor González Ramallo, Martha Kestler Hernández, Amaia Mari Hualde, Mercedes Marín, Manuel Martínez-Sellés, Patricia Muñoz, Cristina Rincón, Hugo Rodríguez-Abella, Marta Rodríguez-Créixems, Blanca Pinilla, Ángel Pinto, Maricela Valerio, Pilar Vázquez, Eduardo Verde Moreno; **Hospital Universitario La Paz**, (Madrid): Isabel Antorrena, Belén Loeches, Alejandro Martín Quirós, Mar Moreno, Ulises Ramírez, Verónica Rial Bastón, María Romero, Araceli Saldaña; **Hospital Universitario Marqués de Valdecilla**, (Santander): Jesús Agüero Balbín, Carlos Armiñanzas Castillo, Ana Arnaiz, Francisco Arnaiz de las Revillas, Manuel Cobo Belaustegui, María Carmen Fariñas, Concepción Fariñas-Álvarez, Rubén Gómez Izquierdo, Iván García, Claudia González Rico, Manuel Gutiérrez-Cuadra, José Gutiérrez Díez, Marcos Pajarón, José Antonio Parra, Ramón Teira, Jesús Zarauza; **Hospital Universitario Puerta de Hierro**, (Madrid): Jorge Calderón Parra, Marta Cobo, Fernando Domínguez, Alberto Fortaleza, Pablo García Pavía, Jesús González, Ana Fernández Cruz, Elena Múñez, Antonio Ramos, Isabel Sánchez Romero; **Hospital Universitario Ramón y Cajal**, (Madrid): Tomasa Centella, José Manuel Hermida, José Luis Moya, Pilar Martín-Dávila, Enrique Navas, Enrique Oliva, Alejandro del Río, Jorge Rodríguez-Roda Stuart, Soledad Ruiz; **Hospital Universitario Virgen de las Nieves**, (Granada): Carmen Hidalgo Tenorio; **Hospital Universitario Virgen Macarena**, (Sevilla): Manuel Almendro Delia, Omar Araji, José Miguel Barquero, Román Calvo Jambrina, Marina de Cueto, Juan Gálvez Acebal, Irene Méndez, Isabel Morales, Luis Eduardo

López-Cortés; **Hospital Universitario Virgen del Rocío**, (Sevilla): Arístides de Alarcón, Emilio García, Juan Luis Haro, José Antonio Lepe, Francisco López, Rafael Luque; **Hospital San Pedro**, (Logroño): Luis Javier Alonso, Pedro Azcárate, José Manuel Azcona Gutiérrez, José Ramón Blanco, Antonio Cabrera Villegas, Lara García-Álvarez, José Antonio Oteo, Mercedes Sanz; **Hospital de la Santa Creu i Sant Pau**, (Barcelona): Natividad de Benito, Mercé Gurguí, Cristina Pacho, Roser Pericas, Guillem Pons; **Complejo Hospitalario Universitario de Santiago de Compostela**, (A Coruña): M. Álvarez, A. L. Fernández, Amparo Martínez, A. Prieto, Benito Regueiro, E. Tijeira, Marino Vega; **Hospital Santiago Apóstol**, (Vitoria): Andrés Canut Blasco, José Cordo Mollar, Juan Carlos Gainzarain Arana, Oscar García Uriarte, Alejandro Martín López, Zuriñe Ortiz de Zárate, José Antonio Urturi Matos; **Hospital SAS Línea de la Concepción**, (Cádiz): García-Domínguez Gloria, Sánchez-Porto Antonio; **Hospital Clínico Universitario Virgen de la Arrixaca** (Murcia): José Mª Arribas Leal, Elisa García Vázquez, Alicia Hernández Torres, Ana Blázquez, Gonzalo de la Morena Valenzuela; **Hospital de Txagorritxu**, (Vitoria): Ángel Alonso, Javier Aramburu, Felicitas Elena Calvo, Anai Moreno Rodríguez, Paola Tarabini-Castellani; **Hospital Virgen de la Salud,** (Toledo): Eva Heredero Gálvez, Carolina Maicas Bellido, José Largo Pau, Mª Antonia Sepúlveda, Pilar Toledano Sierra, Sadaf Zafar Iqbal-Mirza; **Hospital Rafael Méndez,** (Lorca-Murcia):, Eva Cascales Alcolea, Ivan Keituqwa Yañez, Julián Navarro Martínez, Ana Peláez Ballesta; **Hospital Universitario San Cecilio** (Granada): Eduardo Moreno Escobar, Alejandro Peña Monje, Valme Sánchez Cabrera, David Vinuesa García; **Hospital Son Llátzer** (Palma de Mallorca): María Arrizabalaga Asenjo, Carmen Cifuentes Luna, Juana Núñez Morcillo, Mª Cruz Pérez Seco, Aroa Villoslada Gelabert; **Hospital Universitario Miguel Servet** (Zaragoza): Carmen Aured Guallar, Nuria Fernández Abad, Pilar García Mangas, Marta Matamala Adell, Mª Pilar Palacián Ruiz, Juan Carlos Porres; **Hospital General Universitario Santa Lucía** (Cartagena): Begoña Alcaraz Vidal, Nazaret Cobos Trigueros, María Jesús Del Amor Espín, José Antonio Giner Caro, Roberto Jiménez Sánchez, Amaya Jimeno Almazán, Alejandro Ortín Freire, Monserrat Viqueira González; **Hospital Universitario Son Espases** (Palma de Mallorca): Pere Pericás Ramis, Mª Ángels Ribas Blanco, Enrique Ruiz de Gopegui Bordes, Laura Vidal Bonet; **Complejo Hospitalario Universitario de Albacete** (Albacete): Mª Carmen Bellón Munera, Elena Escribano Garaizabal, Antonia Tercero Martínez, Juan Carlos Segura Luque; **Hospital Universitario Terrassa:** Cristina Badía, Lucía Boix Palop, Mariona Xercavins, Sónia Ibars. **Hospital Universitario Dr. Negrín** (Gran Canaria): Eloy Gómez Nebreda, Ibalia Horcajada Herrera, Irene Menduiña Gallego. **Complejo Hospitalario Universitario Insular Materno Infantil** (Las Palmas de Gran Canaria): Héctor Marrero Santiago, Isabel de Miguel Martínez, Elena Pisos Álamo. **Hospital Universitario 12 de Octubre** (Madrid): Carmen Díaz Pedroche, Fernando Chaves, Santiago de Cossío, Francisco López Medrano, Mª Jesús López, Javier Solera, Jorge Solís. **Hospital Universitari Bellvitge** (Barcelona): Carmen Ardanuy, Guillermo Cuervo Requena, Sara Grillo, Alejandro Ruiz Majoral.
